# Absence of the Min System Does Not Cause Major Cell Division Defects in *Agrobacterium tumefaciens*

**DOI:** 10.3389/fmicb.2018.00681

**Published:** 2018-04-09

**Authors:** Sue A. Flores, Matthew Howell, Jeremy J. Daniel, Rebecca Piccolo, Pamela J. B. Brown

**Affiliations:** Division of Biological Sciences, University of Missouri, Columbia, MO, United States

**Keywords:** *Agrobacterium*, cell division, Min system, FtsZ, chromosome segregation

## Abstract

In *A. tumefaciens*, the essential FtsZ protein is located at the growth pole before shifting to the mid-cell right before division. Loss of FtsZ causes a halt in cell separation and lysis of cells. To understand how FtsZ polymerization is regulated to properly localize the FtsZ ring at the mid-cell, we have conducted a systematic characterization of the Min system in *A. tumefaciens*. Our findings indicate that the Min system is not required for cell survival. Yet, we find that the deletion of either *minE* or *minCDE* results in a broad cell size distribution, including an increase in the proportion of short and long cells. We observe that the site of constriction is misplaced in the *minE* or *minCDE* deletion strains allowing for short cells to arise from sites of constriction near the cell poles. Remarkably, the short cells are viable and contain DNA. In order to observe chromosome replication and segregation in these strains, YFP-ParB is used as a proxy to track the origin of replication as cells elongate and divide. In the absence of the Min proteins, duplication and segregation of the origin of replication is frequently delayed. Taken together, our data suggest that the Min system contributes to the proper regulation of FtsZ placement and subsequent cell division. Furthermore, the failure to precisely place FtsZ rings at mid-cell in the *min* mutants impacts other cell cycle features including chromosome segregation.

## Introduction

Most bacteria precisely place the site of cell division at or near mid-cell through proper positioning of FtsZ to initiate divisome assembly. FtsZ forms a ring-like structure at or near the mid-cell along the cytoplasmic surface of the inner membrane (Bi and Lutkenhaus, 1991). FtsZ is stabilized along the membrane by interactions with FtsA leading to the formation of FtsZ filaments (Szwedziak et al., 2014). The FtsZ filaments form a ring-like structure at the future site of cell division and guide other divisome proteins, including peptidoglycan synthases, to the mid-cell (Ma et al., 1996; Sun and Margolin, 1998; Den Blaauwen et al., 1999; Li et al., 2007; Goley et al., 2011). The order of recruitment and essentiality of individual cell division proteins varies across species, but the general mechanism of cell division appears to be broadly conserved (Lutkenhaus and Du, 2017). After assembly of the cell division machinery is complete, peptidoglycan biosynthesis is activated at mid-cell (Addinall et al., 1997; Müller et al., 2007; Möll and Thanbichler, 2009). As the ring constricts, septal peptidoglycan is synthesized inwards to build the new poles of the daughter cells. Septal peptidoglycan synthesis requires the monofunctional PBP3, the SEDS protein FtsW, and the bifunctional PBP1b (Botta and Park, 1981; Bertsche et al., 2006; Cho et al., 2016). The GTPase-dependent treadmilling motion of the FtsZ filaments drives the movement of the peptidoglycan biosynthesis machinery around the circumference of the cell division site, enabling the synthesis of concentric rings of peptidoglycan to form the septum (Bisson-Filho et al., 2017; Yang et al., 2017).

How is FtsZ properly positioned at mid-cell? There are several well characterized mechanisms to ensure proper positioning of the FtsZ rings (for review see Wu and Errington, 2011; Rowlett and Margolin, 2015). In rod-shaped bacteria such as *Escherichia coli* and *Bacillus subtilis*, the Min system and nucleoid occlusion (NO) prevent FtsZ rings from forming near cell poles or over nuceloids, respectively. In *E. coli*, the MinCDE proteins rapidly oscillate from pole to pole guiding FtsZ to the mid-cell (Hu and Lutkenhaus, 1999; Raskin and de Boer, 1999a,b; Fu et al., 2001; Hale et al., 2001). Since MinC is an inhibitor of FtsZ-ring assembly (Hu and Lutkenhaus, 2000; Justice et al., 2000; Dajkovic et al., 2008), FtsZ rings only form in mid-cell regions where the concentration of MinC is low. MinC forms a complex with MinD, an ATPase which dimerizes and binds to the cell membrane when bound to adenosine triphosphate (ATP) (de Boer et al., 1991, 1992; Hu and Lutkenhaus, 2003). MinE binds to MinD-ATP causing ATP hydrolysis and release of the MinCD complex from the membrane (Hu and Lutkenhaus, 2001). Remarkably, MinE can remain bound to the membrane at the pole to dislodge additional MinCD complexes (Loose et al., 2011; Park et al., 2011, 2017). Meanwhile, MinD-ATP is regenerated and cooperatively binds the membrane at the opposite pole. After removing the MinCD complexes from one cell pole, MinE will travel to the opposite pole. Thus, MinE chases MinCD from pole to pole giving rise to regular oscillations. The oscillation of the Min proteins results in a minimum of FtsZ inhibitory activity at mid-cell (Hale et al., 2001; Bonny et al., 2013).

When the genes encoding the Min proteins in *E. coli* are removed simultaneously or individually, cell division and survival is adversely impacted. In the absence of MinE, the cells usually cannot divide and the cells form long, smooth filaments. The block in cell division occurs because inhibition of FtsZ polymerization by MinC occurs throughout the cell (de Boer et al., 1989; Hu and Lutkenhaus, 2000). In the absence of MinC, or its activator MinD, a broad distribution of cell lengths is observed (de Boer et al., 1989). Both mini cells and long filaments are observed since FtsZ polymerization can occur at the cell poles or near mid-cell leading to asymmetric cell division events. FtsZ polymerization is restricted to poles and mid-cell in the absence of the Min system due to the presence of the nucleoid occlusion protein, SlmA (Bernhardt and de Boer, 2005). The FtsZ inhibitory activity of SlmA is activated by binding specific sites on the DNA near the origin of replication (Cho et al., 2011; Tonthat et al., 2011). Thus, as DNA replication is completed and the origins segregate to the cell poles, a minimal inhibitory zone is formed at mid-cell. SlmA binding to DNA activates its ability to bind the C-terminal tail of FtsZ causing depolymerization of FtsZ filaments (Du and Lutkenhaus, 2014). Under nutrient rich conditions, loss of the Min system and nucleoid occlusion is synthetically lethal; however under nutrient limited conditions the cells continue to grow and divide relatively well (Bernhardt and de Boer, 2005). When both the Min proteins and SlmA are absent, FtsZ ring placement is more accurate than in cells with only SlmA suggesting that other mechanisms contribute to the proper placement of FtsZ-ring in the absence of both Min proteins and SlmA (Bailey et al., 2014; Cambridge et al., 2014).

Indeed, the Min system is not universally distributed among bacteria suggesting the existence of alternative mechanisms of FtsZ positioning. MinCD is present in diverse bacteria, MinE is found in a more restricted range of bacteria, and other bacteria do not contain a Min system (Rothfield et al., 2005). For example, the Caulobacterales clade of alphaproteobacteria do not contain obvious homologs of the Min proteins. Furthermore, in *Caulobacter crescentus*, a bacterial model system within the Caulobacterales, only the final step of cell division takes place after completion of chromosome segregation suggesting that nucleoid occlusion does not occur (Jensen, 2006). Instead, *Caulobacter cresentus* uses at least two distinct mechanisms for regulation of cell division (Thanbichler and Shapiro, 2006; Radhakrishnan et al., 2010; Kiekebusch et al., 2012). MipZ is a distinct member of the MinD/ParA family of ATPases that contribute to spatial organization with bacterial cells (Lutkenhaus, 2012). MipZ forms a bipolar gradient on the nucleoid by binding to DNA sites near the origin of replication and directly interacts with FtsZ, inhibiting filament formation near the cell poles (Thanbichler and Shapiro, 2006; Kiekebusch et al., 2012). KidO is an NAD(H)-binding oxidoreductase that provides temporal regulation of FtsZ-ring assembly (Radhakrishnan et al., 2010). KidO binds FtsZ and prevents premature filament assembly at mid-cell. KidO is proteolytically cleared from the cell during elongation and the initiation of cell division, enabling efficient FtsZ-ring formation at mid-cell. KidO reappears late during cell division and is recruited to the mature divisome where it likely contributes to FtsZ-ring disassembly during constriction. Thus, together MipZ and KidO restrict FtsZ-ring formation to the mid-cell of predivisional cells.

Remarkably, not all alphaproteobacterial species lack a Min system. Among the alphaproteobacteria, the MinCDE proteins are found among the Rhodospirallales, Rhodobacterales, and Rhizobiales clades. The *minCDE* cluster is likely regulated by CtrA, the master cell cycle regulator, in several Rhizobiales species including *Brucella abortus* (Bellefontaine et al., 2002), *Prosthecomicrobium hirschii* (Williams et al., 2016), and *Sinorhizobium meliloti* (Pini et al., 2015). In *S. meliloti*, CtrA negatively regulates *minCD* expression (Pini et al., 2015) and overexpression of MinCD inhibits cell division (Cheng et al., 2007). Together, these observations suggest that the Min system may contribute to the regulation of cell division in the Rhizobiales. Here, we expand our knowledge about the function of the Min system in Rhizobiales by characterizing its contribution to regulation of cell division in *Agrobacterium tumefaciens*. The *A. tumefaciens* genome reveals the presence of an operon predicted to encode the MinCDE proteins, but there is not an obvious nucleoid occlusion system or MipZ homolog (Curtis and Brun, 2014). The MinCDE proteins from *Agrobacterium* share significant sequence similarity with the *E. coli* proteins (percent identities: MinC 31.70%, MinD 61.05%, and MinE 39.29%) suggesting that they may have conserved functions. In this work, we have systematically characterized the role of the Min proteins on cellular morphology, constriction site placement, and chromosome segregation. Our results suggest that the Min system contributes to the regulation of cell division; however, other FtsZ-positioning proteins likely exist in *A. tumefaciens*.

## Materials and methods

### Bacterial strains and culture conditions

A list of all bacterial strains and plasmids used in this study is provided in Table 1. *A. tumefaciens* C58 and derived strains were grown in AT minimal media with 0.5% glucose (ATGN) (Morton and Fuqua, 2012b) without exogenous iron at 28°C with shaking. For *sacB* counterselection during construction of deletion mutants 5% sucrose replaced glucose as the sole carbon source (ATSN). *E. coli* DH5α and S17-1 λ *pir* were routinely cultivated in Luria-Bertani (LB) medium at 37°C with shaking. When appropriate, kanamycin was used at 300 μg/ml for *A. tumefaciens* and 50 μg/ml for *E. coli*. When indicated, IPTG was used as an inducer at a concentration of 1 mM.

**Table 1:** Bacterial strains and plasmids used in this study.

**Strain or plasmid**	**Relevant characteristics**	**References/Source**
**PLASMIDS**		
pNTPS138/139	Km^r^; Suicide vector containing *oriT* and *sacB*	D. Alley
pNTPS139Δ*minC*	Km^r^ Suc^s^; deletion plasmid for *minC*	This study
pNTPS139Δ*minD*	Km^r^ Suc^s^; deletion plasmid for *minD*	This study
pNTPS139Δ*minE*	Km^r^ Suc^s^; deletion plasmid for *minE*	This study
pNTPS139Δ*minCDE*	Km^r^ Suc^s^; deletion plasmid for *minCDE*	This study
pRV-Pvan-FtsZ-GFP	Km^r^; replicating plasmid for constitutive expression of FtsZ-GFP	Howell et al., 2017a
pSRKKm-Plac-YFP-ParB	Km^r^; replicating plasmid for inducible expression of YFP-ParB	Ehrle et al., 2017
***E. COLI STRAINS***		
DH5α	Cloning strain	Life Technologies
S17-1	Sm^r^; RP4-2, Tc::Mu,Km-Tn7, for plasmid mobilization	Simon et al., 1983
***A. TUMEFACIENS STRAINS***		
C58	Nopaline type strain; pTiC58; pAtC58	Watson et al., 1975
C58 Δ*minC*	C58 with deletion of *minC*	This study
C58 Δ*minD*	C58 with deletion of *minD*	This study
C58 Δ*minE*	C58 with deletion of *minE*	This study
C58 Δ*minCDE*	C58 with deletion of *minCDE*	This study

### Plasmid construction

PCR was used to amplify ~ 500 bp of flanking sequence upstream (primers 1 and 2) and downstream (primers 3 and 4) of the gene targeted for deletion. Primers used for amplification of regions upstream and downstream of *minC* (Atu 3249), *minD* (Atu3248), *minE* (Atu3247) are shown in Table 2. For regions upstream and downstream of the *minCDE* locus, primers Atu 3249 P1-Spel, Atu 3249 P2, Atu 3247 P3, and Atu 3247 P4-BamHI were used. All PCR reactions contained 10 μM of each primer, 100 ng of genomic DNA purified from wildtype *A. tumefaciens* C58, 10 mM deoxynucleotides (dNTPs; New England Biolabs), 0.5 U Phusion DNA Polymerase (Thermo scientific), 1.5% DMSO, and 5X Phusion GC Buffer (Thermo Scientific). Upstream and downstream DNA fragments were amplified by routine PCR reactions with the following cycling conditions: denaturation 98°C for 30 s, annealing 2° higher than the calculated annealing temperature of the primers for 30s, extension 72°C for 30 s, and final extension was done at 72°C for 5 min. The PCR ran for 30 cycles. PCR products were run on a 0.8% agarose gel by electrophoresis, stained with DNA SafeStain (Midwest Scientific) and gel purified using GeneJet PCR purification kit (Thermo Scientific). A second PCR reaction was done using PCR SOEing (synthesis by overlap extension) to anneal linker sequence from the 500 bp upstream and downstream together as described previously (Merritt et al., 2007). Briefly, purified PCR products were used as both templates and primers for a five-cycle PCR. A final PCR step with primers 1 and 4, using 2 μl of the second-step reaction mix as the template, generates the full-length spliced product. PCR products were then gel purified. PCR products and the pNTPS139 vector were then digested overnight at 37°C using enzymes Spel and BamHl (New England Biolabs). Digested products were gel purified and ligated together using T4 DNA ligase (New England Biolabs). Ligations were transformed into *E.coli* DH5α competent cells using the suggested manufacturer protocol (Invitrogen). Transformants were plated on LB-agar plates containing kanamycin. Individual colonies from the transformation where then grown up overnight in LB with Kan50 and plasmid extraction was done using GenJet plasmid miniprep kit (Thermo Scientific). Plasmid inserts were verified by sequencing and the plasmids were transformed into *E. coli* S-17 by electroporation.

**Table 2:** Synthesized DNA primers used in this study.

**Primers**	**Sequence**
Atu 3249 P1-Spel	5′-ACA CGT ACT AGT CAG GCC GAT GCG G−3′
Atu 3249 P2	5′-AAG CTT GGT ACC GAAA TTC GCG AAG CTC G−3′
Atu 3249 P3	5′-GAA TTC GGT ACC AAGCTT CGA ACGCTG G−3′
Atu 3249 P4-BamHI	5′-CGC GCG GGA TCC GCA ATC GAA TTG ACC A−3′
Atu 3248 P1-Spel	5′-ACA CGT ACT AGT TAT GGC CTG ATG CTG C−3′
Atu 3248 P2	5′-AAG CTT GGT ACC GAA TTC AGG AGG GCT G−3′
Atu 3248 P3	5′-GAA TTC GGT ACC AAG CTT TAC AAC GAC TA−3′
Atu 3248 P4-BamHI	5′-CGC GCG GGA TCC GTT CGC CCG TCG ATG A−3′
Atu 3247 P1-Spel	5′-ACA CGT ACT AGT GCC GAT CTT GCC GGG C−3′
Atu 3247 P2	5′-AAG CTT GGT ACC GAA TTC CTG CGC GCT−3′
Atu 3247 P3	5′-GAA TTC GGT ACC AAG CTT GAT GCT CAT GC−3′
Atu 3247 P4-BamHI	5′-CGC GCG GGA TCC GAA TGG GTC ATC GCC G−3′
MinCDE P5	5′-CAT GGA ATG CGT GGC GAG CAC GAA TAC G−3′
MinCDE P6	5′-GAA GCC CGC ATG CCA TAGG ATA CGT TGC AG−3′

### Deletion of target genes in *A. tumefaciens*

Nonpolar, markerless deletions of the *A. tumefaciens* individual *minC* (Atu 3249), minD (Atu 3248), and minE (Atu 3247) genes and the entire locus were generated using the plasmids pNPTS139Δ*minC*, pNPTS139Δ*minD*, pNPTS139Δ*minE, and* pNTPS139Δ*minCDE* following an established protocol (Morton and Fuqua, 2012a). Deletion of target genes was confirmed by colony PCR using the indicated primer pairs: Δ*minC* (Atu 3249 P1-Spel and MinCDE P6), Δ*minD* (Atu 3248 P1-Spel and Atu 3249 P4-BamHI), Δ*minE* (Atu 3247 P1-Spel and Atu 3248 P4-BamHI), and Δ*minCDE* (MinCDE P5 and Atu 3249 P4-BamHI). PCR products were gel purified and sequence verified to confirm deletion of the target gene.

### Growth curve analysis

Strains were grown in ATGN until exponential phase was reached, then back diluted to reach an OD_600_ = 0.1 in 1 ml of ATGN. 200 μl of culture was placed into 3 wells of a 96-well plate. A BioTek Synergy H1 Hybrid Reader was programed to read the optical density at 600 nm every 5 min after shaking for 1 min. The plate reader was maintained at a temperature of 28°C for a period of 36 h. Growth curve experiments were completed in triplicate and a total of four biological replicates were completed.

### Cell viability assays

Serial dilutions of *A. tumefaciens* cells were spotted on ATGN plates to assess the viability of the *min* mutants. Exponential cultures (OD_600_ = 0.4–0.6) were diluted to OD_600_ 0.05 in ATGN. Cells were then serially diluted and 4 μl of each dilution was spotted onto ATGN plates. Plates were grown for 3 days at 28°C and imaged.

### Phase contrast microscopy, cell length analysis, and quantitation of constriction position

Cells were grown in ATGN media until exponential phase was reached. A small volume (0.6–0.8 μl) of live cells were then placed onto a 1% agarose ATGN pad as described previously (Brown et al., 2012; Howell et al., 2017b). Phase contrast microscopy was performed with an inverted Nikon Eclipse TiE with a QImaging Rolera em-c^2^ 1 K EMCCD camera and Nikon Elements Imaging Software. Cell length distributions of 948 cells per strain were determined using the longest medial axis measured using MicrobeJ software (Ducret et al., 2016). Sites of cell constrictions were determined for ~1,000 individual cells for each strain using stacked phase contrast images. Sites of constriction were autodetected using a preset MicrobeJ constriction detection function. To determine the polar orientation of each cell, old poles were identified using the lipophilic dye FM4-64 as previously described (Howell et al., 2017b).

### Fluorescence microscopy

For DNA staining, 1 ml of cells at an OD_600_ = 0.4–0.6 were treated with 1 μl of Sytox Orange (Invitrogen) for 5 min in the dark. Cells were pelleted and washed with PBS two times to remove excess dye. Cell pellets were resuspended in PBS and cells were imaged immediately on an agarose pad. Replicating plasmids pRV-Pvan-FtsZ-GFP and pSRKKm-Plac-YFP-ParB were introduced in wildtype cells and *min* mutants via an established electroporation protocol (Morton and Fuqua, 2012a). For imaging of YFP-ParB, exponential phase cells were diluted to OD_600_ = 0.2 and were induced with IPTG for 4 h until reaching an OD_600_ = 0.4–0.6 and were then imaged on agarose pads. Dual channel images were stacked and cell outlines and YFP-ParB foci were automatically detected using MicrobeJ (Ducret et al., 2016). Demographs depicting YFP-ParB localization were created by capturing the fluorescent intensity along the midline of the longitudinal axis of each cell and ordering these images by cell length. Both cell outlines and identified YFP-ParB foci were manually reviewed to ensure that the plots reflect accurate sites of YFP-ParB foci. For imaging of cells expressing constitutive FtsZ-GFP, cells were grown to OD_600_ = 0.4–0.6 and placed on agarose pads. Cells were imaged using phase contrast and epifluorescence microscopy with the appropriate filters. For timelapse microscopy cells were grown on ATGN 1.5% agarose pads with images collected either every 5 or 10 min.

## Results

### Deletion of *min* genes does not have a large impact on cell growth or viability, but causes a broader cell length distribution

**Figure 1:**
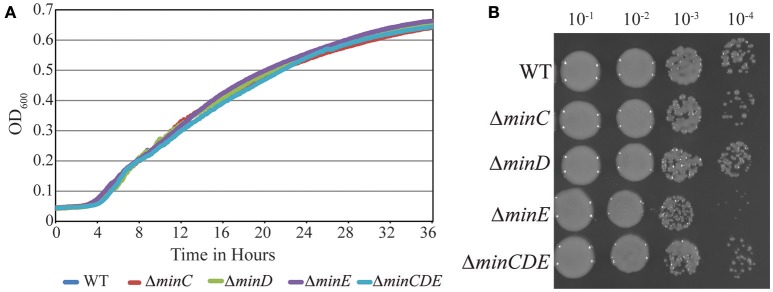
Growth and viability of *min* mutants is not compromised. **(A)** Growth of Wildtype *A. tumefaciens* strain C58 and *min* mutants is monitored over 36 h by observing the increase in optical density. **(B)** Exponentially growing cells were diluted to OD _600_ = 0.05 and serial dilutions were spotted on nutrient rich medium to observe viability.

**Figure 2:**
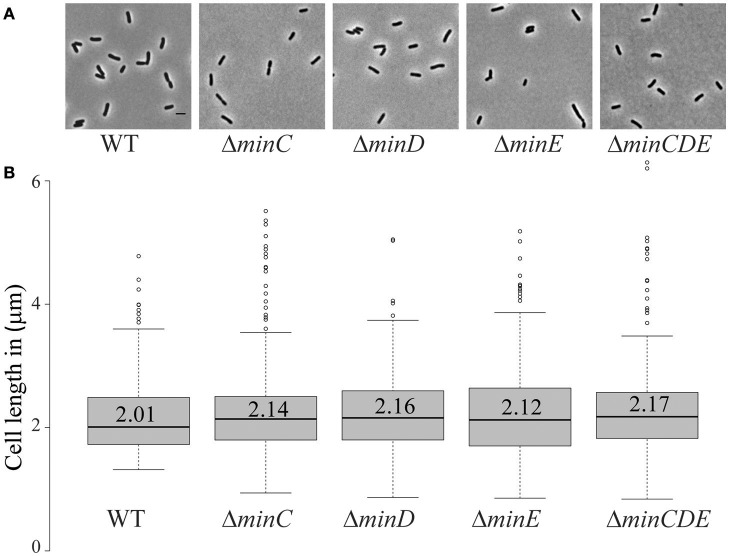
Short cells accumulate in *min* mutants. **(A)** Phase contrast images of representative wildtype and *min* mutant cells. Scale bar = 2 μm. **(B)** Box plots illustrate cell length distributions of wildtype cells and *min* mutants. Medians are shown by the labeled center lines. Box limits indicate the 25th and 75th percentiles as determined by R software; whiskers extend 1.5 times the interquartile range and outliers are represented by dots. Cell lengths measured from 948 cells for each strain.

**Figure 3:**
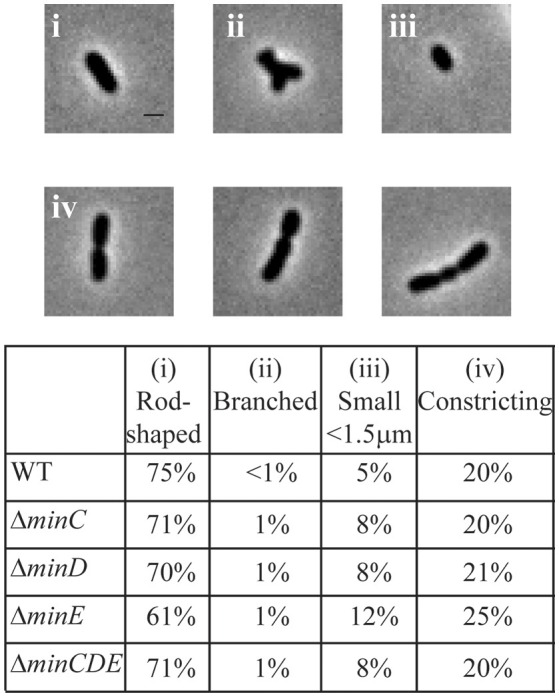
Atypical morphologies are observed in *min* mutants. Phase contrast images of representative morphologies. All images taken from the Δ*minE* cell population. Scale bar = 1 μm. (**top**) Quantitation of bacterial morphologies. (**bottom**) The percentage of cells with a rod-shaped morphology (i: 1.5–3.5 μm in length), branched morphology (ii), short cell morphology (iii: <1.5 μm in length), and cells with visible constrictions (iv) are shown in the table. Cell morphologies were categorized from at least 948 cells for each strain.

Transposons accumulate in the *min* region of *A. tumefaciens* during saturating transposon mutagenesis experiments in *A. tumefaciens* suggesting that the Min system is not required for cell survival (Curtis and Brun, 2014). To verify these results, we constructed markerless deletions in each *min* gene and the entire *min* locus. Growth curves of *min* mutants were indistinguishable from the growth curve of the parent strain (Figure 1A). The *min* mutants are viable (Figure 1B) with only a slight decrease in viability in Δ*minE*, suggesting that unregulated MinCD is more problematic than loss of the entire *min* locus. Although the *min* mutants are viable, phase contrast images of the cell populations revealed a small but consistent proportion of cells with atypical morphologies, including short cells (Figure 2A). Quantitative image analysis was used to determine the cell length distributions of the *min* mutants (Figure 2B). While the medians of the cell length distributions of the *min* mutants are actually slightly longer than that of the parent strain, the length of the whiskers is significantly increased suggesting that both short and long cells are accumulating in the *min* mutants. Next, we determined the percentage of cells with typical cell lengths (defined as 1.5–3.5 μm), cells with branches or bulges, short cells (< 1.5 μm), and cells with visible constrictions (Figure 3). Indeed, these observations confirm the presence of a small but reproducible proportion of short cells in the *min* mutant cells (Figure 3, panel iii). Furthermore, although the overall proportion of cells with constrictions is not impacted by the loss of individual *min* genes or the entire locus, we observe an increase in cells with obvious asymmetric constrictions or multiple constrictions (Figure 3, panel iv).

### The Min system contributes to precise placement of constriction sites

**Figure 4:**
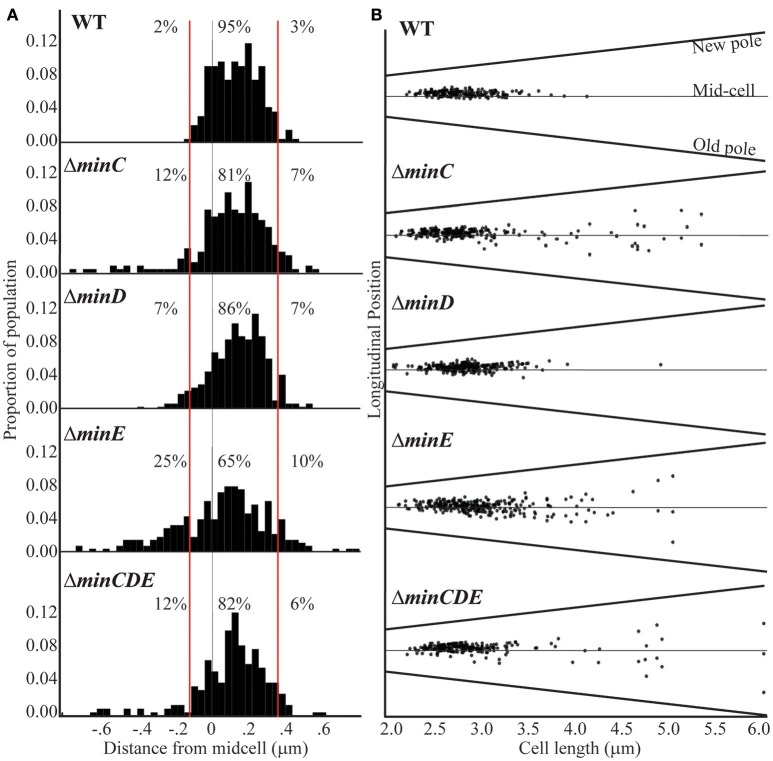
Cell constriction placement in wildtype and *min* mutant cells. **(A)** Histograms of constriction position relative to distance from the midcell (gray line; relative position = 0). The red lines at −0.1 and 0.35 μm mark the region where constrictions typically form in wildtype cells. Negative numbers are closer to the old pole whereas the positive numbers are closer to the new pole. Percentages indicate the proportion of constrictions placed farther than −0.1 μm from mid-cell, placed between −0.1 and 0.35 μm, and placed more than 0.35 μm from mid-cell. **(B)** The longitudinal position of constrictions is plotted against cell length. For this analysis, cells are ordered by cell length and black dots indicate the position of the constriction. Mid-cell is indicated by the center gray line and the slanted lines indicate the position of the new and old pole for each cell. For these analysis, the predivisional cells within a population of ~1,000 cells were analyzed (*n* = 186 for wildtype; *n* = 242 for Δ*minC, n* = 262 for Δ*minD, n* = 285 for Δ*minE*, and *n* = 225 for Δ*minCDE*).

The previous observations suggest that the Min system may contribute to the proper placement of the site of cell division in *A. tumefaciens*. To better understand the function of the Min system in the establishment of constriction sites, we quantitated the position of constriction sites in wildtype cells (*n* = 186). In this analysis, the true-mid cell is defined as 0 and negative values indicate positions of constrictions closer to the old pole whereas positive values indicate positions of constrictions closer to the new pole. Using the wildtype data set, we define a typical constriction placement to occur near mid-cell with a bias toward the new pole: 95% of constrictions formed between −0.1 and 0.35 μm from the true mid-cell position (Figure 4A, left). In addition, constrictions are consistently observed in cells with lengths between 2.5 and 3.5 μm (Figure 4A, right). These observations indicate that the site of constriction is established well before the cell has completed elongation. Cells longer than 4 μm in length with constrictions are rarely observed, presumably because the cells have successfully completed cell division.

In the *min* mutants, constrictions form in ~20% of the cell population which is similar to what is observed in wildtype (Figure 3); however, the placement of the constrictions is perturbed. The *min* mutants have a broader distribution of constriction placement (Figure 4A). The Δ*minE* mutant in particular has lost the ability to maintain the proper bias of constriction site placement near the new pole. In contrast, when the entire *min* locus is absent 82% of constrictions are observed in the typical position and the bias for constriction placement toward the new pole is retained. These observations suggest that the unregulated activity of MinCD results in a more random positioning of constrictions, but proper positioning of constrictions is frequently retained when the *min* locus is entirely deleted. Although constriction sites form more randomly in *min* mutants, we rarely observe constriction sites immediately adjacent to the cell poles (Figure 4B) and short cells, rather than mini cells are formed. Since constriction sites are not biased only toward the new pole in *min* mutants, this phenotype cannot be readily explained by the continuation of growth at the new pole and may indicate the existence of another mechanism to protect the poles. Furthermore, an increase in long cells (>4 μm) with visible constrictions (Figure 4B) is observed in *min* mutants. This observation suggests that cell division efficiency is adversely impacted in the *min* mutants. It is possible that the perturbations of the Min system result in a delay in cell division or an increase in the frequency of constrictions that do not lead to a productive cell division event.

### FtsZ-rings form in *min* mutants

**Figure 5:**
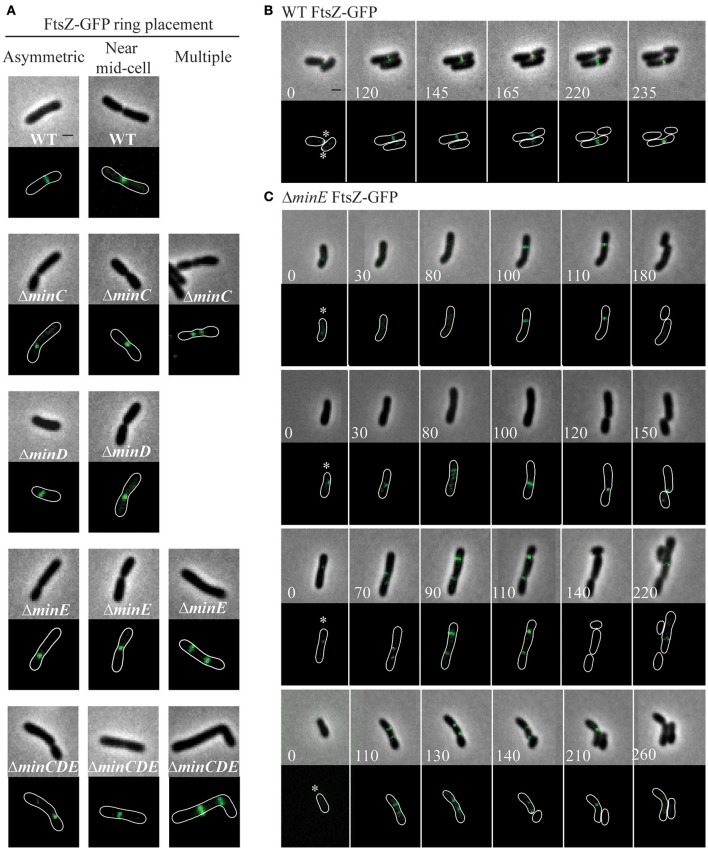
FtsZ-GFP ring position in wildtype and *min* mutant cells. **(A)** Representative images of FtsZ-GFP localization patterns in wildtype and *min* mutants including asymmetric localization, near mid cell localization, and multiple ring formation. The absence of an image indicates that the localization pattern is not observed in the strain. **(B)** Timelapse microscopy showing typical FtsZ-GFP localization patterns in wildtype cells. Asterisks mark the growing poles. **(C)** Timelapse microscopy of FtsZ-GFP localization in Δ*minE* cells. Asterisks mark the growing poles. Images shown in panels **B** and **C** are at the same scale. Scale bars = 1 μm.

In order to provide additional insights into the position of constriction placement, we introduced a plasmid which constitutively expresses *ftsZ-gfp* at a low level (Howell et al., 2017a) into wildtype and *min* mutant cells. In wildtype cells, FtsZ-rings initially form at an asymmetric position near the new pole and mark the future site of cell division (Figures 5A,B, Movie S1). As the cell continues to elongate at the new pole, the FtsZ ring is ultimately positioned near mid-cell and constriction leads to the appearance of a discrete focus of FtsZ in late predivisional cells. In the *min* mutants, FtsZ rings are observed at asymmetric positions, near mid-cell and in some cells multiple FtsZ rings form (Figure 5A). Unlike the pattern of FtsZ ring formation in wildtype cells which is very consistent, the pattern of FtsZ ring formation is variable in the *min* mutants. For example, asymmetric FtsZ rings form in positions biased toward either the new or old pole in Δ*minE* mutants (Figure 5C, top two panels, Movies S2, S3). In either case, the establishment of an FtsZ ring can lead to a cell division event which creates daughter cells of different cell sizes. These observations are consistent with the broader cell length distribution of the *min* mutants which includes both short and long cells (Figure 2) and with the asymmetric positioning of constrictions (Figure 4). In addition, Δ*minC*, Δ*minE*, and Δ*minCDE* cells with multiple FtsZ rings are readily observed (Figure 5A). In Δ*minE* cells with two FtsZ rings, both sites marked with FtsZ undergo constriction leading to the production of a bow-tie morphology and cell division ultimately occurs at either both or a single site marked by an FtsZ ring (Figure 5C, bottom two panels, Movies S4, S5).

### Cell division is initiated prior to nucleoid separation

**Figure 6:**
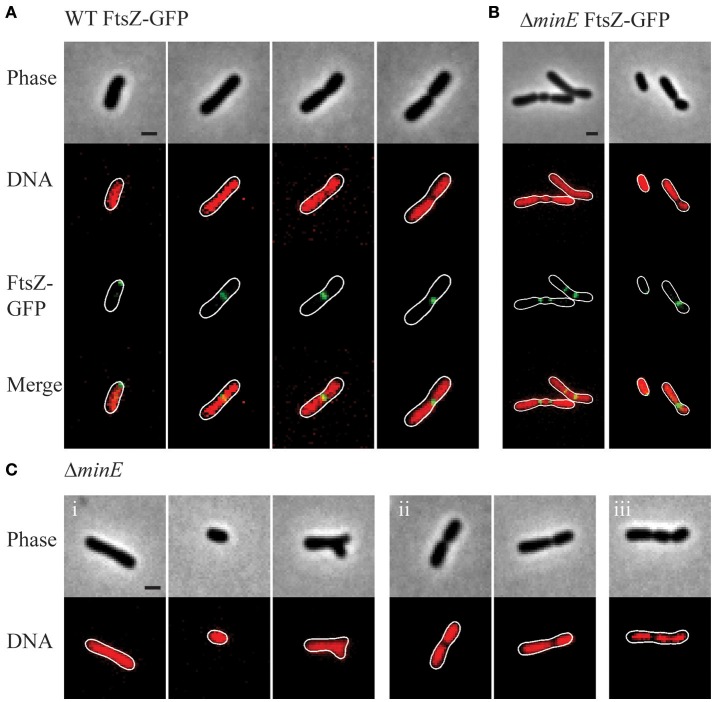
Nucleoid position in wildtype and *min* mutant cells. **(A)** Representative images demonstrating FtsZ-GFP localization in wildtype cells with Sytox Orange stained DNA. **(B)** Representative images of FtsZ-GFP localization in Δ*minE* cells with Sytox Orange stained DNA. **(C)** Representative images from the Δ*minE* mutant population. Sytox Orange was used to label DNA and observe localization patterns including (i) diffuse, (ii) separated nucleoids, and (iii) other patterns. Scale bars = 1 μm.

After observing that FtsZ rings are present in wildtype cells without visible constrictions (Figure 5B), we next aimed to determine if FtsZ rings form over DNA (Figure 6). In elongating wildtype cells, FtsZ is typically observed either as a polar focus or in an asymmetric FtsZ ring, whereas Sytox Orange labeling indicates that the DNA is diffuse (Figure 6A, columns 1-2). The DNA remains diffuse in cells with early constrictions (Figure 6A, column 3) and only forms separated nucleoids in deeply constricted cells (Figure 6A, column 4). These observations are consistent with the possibility that *A. tumefaciens* does not use a nucleoid occlusion mechanism to position FtsZ at mid-cell. Similar results are observed in the *min* mutants, as exemplified by Δ*minE* (Figure 6B). Notably, in the Δ*minE* mutant FtsZ rings form over the top of DNA at asymmetric positions and when multiple FtsZ rings are present. Finally, we observed that short cells in the Δ*minE* population typically contain diffuse DNA (Figure 6B, right column).

In the wildtype cells and each of the *min* mutants, 90–95% of the cell population (based on observations of ~230 cells/strain) has diffuse DNA. In the *min* mutants, DNA is diffuse in cells without constrictions irrespective of cell shape (shown for Δ*minE* in Figure 6C, panel i), including short and branched cells. Two distinct nucleoids are observed in 5–10% of the wildtype and *min* cell populations (shown for Δ*minE* in Figure 6C, panel ii). As expected due to the formation of asymmetric sites of cell constriction, the *min* mutants contain a higher proportion of cells with asymmetrically separated nucleoids. In <1% of the Δ*minC and* Δ*minE* cells, the presence of more than 2 nucleoids is observed (Figure 6C, panel iii).

### Chromosome segregation is delayed in single *min* mutants

**Figure 7:**
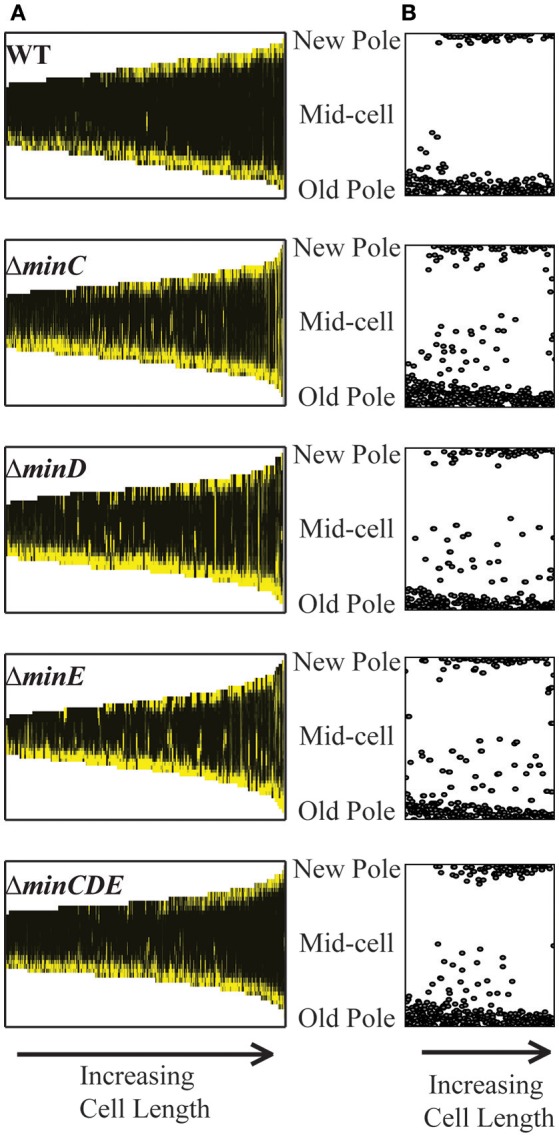
YFP-ParB localization in wildtype and *min* mutant cells. **(A)** Demographs of YFP-ParB localization. Cells are ordered according to cell length and positions of the new pole, mid-cell, and old pole are indicated. **(B)** Position of YFP-ParB foci are plotted relative to longitudinal position in the cells. At least 500 cells were analyzed for each strain.

Since the inefficiency of cell division in the *min* mutants may cause a delay in chromosome segregation, we next tracked the early stages of chromosome segregation by introducing an IPTG inducible plasmid expressing *yfp-parB* (Ehrle et al., 2017) into the *min* mutants. ParB attaches to the *parS* site near the origin of replication, making it suitable to track the movement of newly replicated origin (Ehrle et al., 2017). In *A. tumefaciens* ParB localizes at the old pole and as the cell nears division a second focus appears and rapidly tracks across the cell to the new pole ensuring both cells receive a copy of the chromosome (Figure 7; Ehrle et al., 2017). The longitudinal profile of over 500 cells expressing YFP-ParB were aligned in order of cell length to create a demograph depicting the localization of YFP-ParB throughout the cell cycle (Figure 7A). In wildtype cells, YFP-ParB is observed in three patterns: first, a single focus is observed in short cells, next we observe a brief transition period where a second focus of YFP-ParB appears and transits along the longitudinal axis of the cell, finally in predivisional cells, both foci are anchored at opposite poles. To examine the YFP-ParB localization pattern at the population level, the positions of the YFP-ParB foci were normalized by cell length and plotted along the cell axis (Figure 7B). In wildtype cells, a larger number of foci are observed at the old pole than at the new pole due to the presence of a single focus in short cells and two foci near the old pole at the onset of DNA replication. Notably, very few YFP-ParB foci are observed transiting from old pole to new pole presumably due to the rapid rate of DNA replication and chromosome segregation.

The deletion of single *min* genes results in less consistent patterns of YFP-ParB localization throughout the cell cycle. In the absence of MinC, MinD, or MinE, YFP-ParB is located at a single focus in short cells; however, not all of the foci are found at the old pole (Figure 7A). Furthermore, we observe an increase in the number of foci observed between the poles (Figures 7A,B). This phenotype is due to a combination of cells which accumulate more than 2 YFP-ParB foci and cells in which the duplicated origin is not efficiently transited from pole to pole. Although MinD has been described as a candidate protein to tether DNA to the membrane during chromosome segregation in *E. coli* (Di Ventura et al., 2013), we do not observe a more severe phenotype in the Δ*minD* mutant compared to the Δ*minC* or Δ*minE* mutants. Furthermore, deletion of the entire *min* locus results in a localization pattern of YFP-ParB which is more similar to wildtype. Since the *min* mutants are viable (Figure 1) and even short cells are capable of resuming growth (Figure 5C, bottom panel), we infer that chromosome segregation is delayed presumably due to inefficient cell division in the *min* mutants.

## Discussion

In this work, we have characterized the impact of the Min system on the cell division of *A. tumefaciens*. Similar to findings in *S. meliloti* (Cheng et al., 2007) and consistent with a saturating transposon screen in *A. tumefaciens* suggesting that the *min* genes are not essential (Curtis and Brun, 2014), we have confirmed that the *min* genes are not required for cell viability (Figure 1). Quantitative image analysis of cell morphology reveals that cell length distributions and placement of sites of cell constrictions are perturbed when single *min* genes are deleted compared to wildtype cells (Figures 2–4). In particular, the absence of MinE leads to the accumulation of both long and short cells suggesting that the placement of the septum is perturbed. Indeed, the sites of cell constriction (Figure 4) and localization of FtsZ-GFP is more variable in the Δ*minE* strain (Figure 5) suggesting that the misregulation of MinCD is detrimental to efficient cell division. These observations are consistent with the *E. coli* model of the Min system where MinE regulates the activity of the MinCD complex by driving the oscillation of MinC and MinD from pole-to-pole and preventing the establishment of polar FtsZ rings (Lutkenhaus, 2007; Rowlett and Margolin, 2015). In *A. tumefaciens*, the absence of MinE leads to a more random distribution of active of MinCD complexes, allowing the observed misplacement of FtsZ-rings and constriction sites. Remarkably, asymmetric FtsZ-GFP rings are not observed immediately adjacent to the cell poles in *min* mutants (Figures 5A,C) and can form over DNA in both wildtype cells and *min* mutants (Figure 6). Finally, the absence of the entire Min system has a relatively mild phenotype enabling most cells to divide near mid-cell (Figures 2, 4). Together, these results suggest that other mechanisms for proper placement of FtsZ-rings must exist in *A. tumefaciens*.

While the *A. tumefaciens min* mutant phenotypes are generally consistent with *E. coli* Min model, a key component of this model remains to be tested in *A. tumefaciens*. In *E. coli*, the Min proteins oscillate from pole-to-pole producing a local minimum inhibition zone at mid-cell in which FtsZ-rings can form (Raskin and de Boer, 1999a,b; Meinhardt and de Boer, 2001). In contrast, in *B. subtilis* the MinCDJ system does not use protein oscillation. DivIVA binds to regions of the membrane with negative curvature (Lenarcic et al., 2009; Ramamurthi and Losick, 2009). Next, MinJ acts as an adaptor protein and enables the recruitment of MinD and subsequently MinC to sites of DivIVA localization (Bramkamp et al., 2008; Patrick and Kearns, 2008). Initially, it was thought that the MinCD complexes formed a static bipolar gradient which protects the poles from FtsZ ring assembly (Adams and Errington, 2009; Bramkamp and van Baarle, 2009); however, DivIVA is recruited to the nascent division site when constriction is initiated leading to the formation of DivIVA rings on either side of the division site (Eswaramoorthy et al., 2011). MinCDJ complexes are assembled at these DivIVA rings and presumably prevent the formation of additional FtsZ rings near mid-cell. Based on the presence of *minE* and the absence of *divIVA* and *minJ* in the genome of *A. tumefaciens*, we hypothesize that the MinCD proteins should localize at cell poles and oscillate from pole-to-pole and MinE should exhibit a dynamic localization pattern with enrichment at sub-polar region. Our initial efforts to construct C-terminal fluorescent protein fusions to the *A. tumefaciens* Min proteins have been unsuccessful. A rigorous effort will be needed to observe the localization patterns of the Min proteins in *A. tumefaciens* to determine if pole-to-pole oscillation of the Min proteins contributes to the efficient establishment of constriction sites at the proper position.

In addition to cell division defects, single deletion of *min* genes results in delayed chromosome segregation (Figure 7). At first glance, this finding appears to be consistent with observations of chromosome partitioning defects in *E. coli min* mutants (Akerlund et al., 1992, 2002; Di Ventura et al., 2013); however, there are notable phenotypic differences. First, in *E. coli*, the production of mini-cells devoid of DNA due to polar cell division events is a hallmark of *min* mutants (de Boer et al., 1989). In contrast, short cells arising from misplacement of constriction sites in *A. tumefaciens min* mutants typically contain DNA. This difference may suggest that *A. tumefaciens* does not employ a nucleoid occlusion system to prevent the establishment of FtsZ rings over unsegregated nucleoids (Wu and Errington, 2004; Bernhardt and de Boer, 2005) and is consistent with the absence of an obvious nucleoid occusion protein in the *A. tumefaciens genome* (Goodner et al., 2001; Wood et al., 2001) and the formation of FtsZ-GFP rings over DNA in unconstricted cells (Figure 6A). Second, in *E. coli*, a Δ*minCDE* mutant has a more severe defect in chromosome segregation than a Δ*minC* mutant leading to the hypothesis that MinD may directly contribute to chromosome segregation (Di Ventura et al., 2013). The MinD/ParA family of proteins share an evolutionary history and MinD and ParA function in providing positional information for spatial organization of the FtsZ ring and segregating chromosome, respectively (Lutkenhaus, 2012). *E. coli* MinD can nonspecifically bind chromosomal DNA and may provide polar gradients of DNA tethering sites during chromosome segregation (Di Ventura et al., 2013). If *A. tumefaciens* MinD is capable of binding DNA and tethering chromosomes to the membrane, the predicted random distribution of MinCD complexes in the absence of MinE may explain why the YFP-ParB foci do not rapidly transit from pole-to-pole. Nevertheless, if *A. tumefaciens* MinD is involved in chromosome segregation we would expect to observe segregation defects in the Δ*minD* and Δ*minCDE* strains. Remarkably, the Δ*minC*, Δ*minD*, and Δ*minE* strains exhibit a strikingly similar phenotype with a delay in transition of the YFP-ParB focus from the old pole to the new pole (Figure 7). Furthermore, the Δ*minCDE* strain has a less severe phenotype and YFP-ParB is bipolar in the longest cells. Together, these data suggest that the chromosome segregation defect in the *min* mutants likely arises indirectly as a consequence of less efficient cell division.

Remarkably, in some *C. crescentus* cells lacking MipZ, productive cell division events occur resulting in the production of mini cells which contain DNA (Thanbichler and Shapiro, 2006). Although constrictions form over chromosomes that have not completed segregation, most isolated mini cells contain both an origin of replication and a terminus. These observations suggest that cell division is delayed until DNA replication is finished and the complete chromosome is delivered to the mini cell compartment (Thanbichler and Shapiro, 2006). In most bacteria, DNA replication and chromosome segregation occur simultaneously. These processes consist of three major stages: separation and translocation of the duplicated origin, segregation of the bulk chromosome, and separation of the terminus region (Badrinarayanan et al., 2015; Surovtsev and Jacobs-Wagner, 2018). *C. crescentus* uses the widely distributed ParABS system (Livny et al., 2007) to segregate the *ori* region of the chromosome. Briefly, ParB binds to DNA at the *parS* site which is proximal to the origin of replication. Following duplication of *ori*, one of the ParB-bound *ori* regions remains at the old pole and the other is translocated across the cell to the opposite pole following a receding cloud of ParA (Shebelut et al., 2009; Ptacin et al., 2010; Schofield et al., 2010). ParB is anchored to the poles through a direct interaction with the polar organizing protein PopZ (Bowman et al., 2008; Ebersbach et al., 2008). When PopZ is absent, the chromsomes become untethered from the pole and mini cells without DNA are formed (Ebersbach et al., 2008). MipZ not only inhibits FtsZ-ring assembly, it also binds to ParB, protecting the *ori* proximal regions from FtsZ-ring formation (Thanbichler and Shapiro, 2006). Thus, the processes of cell division and *ori* partitioning are tightly coupled through MipZ. Later stages of cell division and chromosome segregation are also coupled through FtsK. In *C. crescentus*, the N-terminus of FtsK contributes to the stability of FtsZ-rings and the C-terminus of FtsK is responsible for clearing the termini from the division plane (Wang et al., 2006).

The observation that the *A. tumefaciens min* mutants produce short cells that contain DNA may suggest that *A. tumefaciens* also couples the processes of cell division and chromosome segregation. Indeed, the deletion of *popZ* in *A. tumefaciens* results in untethered chromosomes and the production of cells devoid of DNA (Ehrle et al., 2017). In some Δ*popZ* cells, DNA appears to be segregated in the wrong direction across the division plane. FtsK functions as a DNA translocase that assists in the completion of cell division by moving DNA across the division plane in the direction of the termini (Besprozvannaya and Burton, 2014; Badrinarayanan et al., 2015). If a terminus is trapped on the wrong side of the division plane in the absence of PopZ, FtsK may pump DNA in the wrong direction leading to the production of cells without DNA. Notably, whereas the deletion of *popZ* leads to the production of mini cells in *C. crescentus* (Ebersbach et al., 2008), the loss of *popZ* results in the production of a broad distribution of cell lengths in *A. tumefaciens* (Howell et al., 2017a) suggesting that the poles are still largely protected from FtsZ-ring formation. Similarly, when the Min system is removed the short cells sometimes arise (Figure 2) and although *ori* partitioning appears to be delayed (Figure 7), most cells are viable (Figure 1) suggesting that even short cells inherit an intact chromosome. Timelapse microscopy of the Δ*minE* mutant illustrates that short cells are capable of resuming growth following cell division (Figure 5C, bottom panel). Together, these observations suggest that *A. tumefaciens* must use another FtsZ-positioning mechanism to protect the poles and that the processes of DNA replication, chromosome segregation, and cell division must be coordinated.

How might DNA replication, chromosome segregation, and cell division be properly coordinated in *A. tumefaciens*? In *S. meliloti*, expression of *minC* and *minD* was upregulated during depletion of CtrA, implicating this master cell cycle regulator as a transcriptional repressor of this operon (Pini et al., 2015). Remarkably, *minCD* are the only known cell division genes directly regulated by CtrA; however, introduction of a deletion of *minCDE* into the CtrA depletion strain did not rescue the cell division defect suggesting the Min overexpression is not exclusively responsible for the cell division phenotype. Depletion of CtrA in *A. tumefaciens* leads to a block in cell division (Figueroa-Cuilan et al., 2016) and a putative consensus CtrA binding site (TTAA-N_7_-TTAA) is present upstream of *minC* in the *A. tumefaciens* genome. Thus, it is tempting to speculate that the transcription of the *min* genes is under the control of CtrA. Cell-cycle regulation of the Min system may ensure that these proteins are functioning as needed when the cells approach cell division. In *C. crescentus* the expression of *ftsZ* is directly regulated by CtrA (Kelly et al., 1998; Laub et al., 2002) and FtsZ is subject to proteolysis by ClpAP and ClpXP (Williams et al., 2014) leading to cell-cycle variability of FtsZ levels. Even when *ftsZ* is expressed constitutively, it is subject to post-translational control leading to cell cycle variability of FtsZ levels (Williams et al., 2014). The cell cycle variability in FtsZ levels may be a common feature among bacteria with an alphaproteobacterial cell cycle. Thus, in *A. tumefaciens* the cell cycle regulation of the Min system may temporally coordinate the expression levels of the Min and FtsZ proteins. Future studies will be necessary to determine if FtsZ, other divisome components, and the Min proteins are coordinated through cell-cycle regulation in *A. tumefaciens*. Such studies are necessary to better understand how the processes of DNA replication, chromosome segregation, and cell division are coordinated in *A. tumefaciens*.

Overall these results suggest that while the *A. tumefaciens* Min system contributes to the precise positioning of an FtsZ-ring and constriction site near mid-cell, other mechanisms must exist to ensure proper spatial organization during cell division. In *A. tumefaciens*, the phenotype of the Δ*minCDE* strain is milder than that of the individual Δ*minC*, Δ*minD*, or Δ*minE* strains suggesting that this FtsZ positioning system is dispensable for the completion of cell division. There are a number of alternative FtsZ positioning proteins including nucleoid occlusion proteins, MipZ in *C. crescentus* which forms a bipolar gradient and directly inhibits FtsZ-ring assembly near the poles (Thanbichler and Shapiro, 2006; Kiekebusch et al., 2012), and positive regulators which localize to mid-cell and promote FtsZ-ring assembly (Rowlett and Margolin, 2015). Other than the *min* locus, genes encoding candidate FtsZ-positioning proteins cannot be readily identified in the *A. tumefaciens* genome (Goodner et al., 2001; Wood et al., 2001). Thus, further studies of *A. tumefaciens* cell division are likely to reveal novel strategies to ensure proper mid-cell assembly of the divisome.

## Author contributions

SF, MH, JD, and PB designed the experiments. SF, MH, JD, and RP conducted the experiments. SF, MH, RP, and PB analyzed the data. All authors contributed to writing and editing of the manuscript.

### Conflict of interest statement

The authors declare that the research was conducted in the absence of any commercial or financial relationships that could be construed as a potential conflict of interest.
