# Supplementary material for: Absence of the Min System Does Not Cause Major Cell Division Defects in Agrobacterium tumefaciens
Source: Front Microbiol. 2018 Apr 9;9:681. doi: 10.3389/fmicb.2018.00681 (PMC5900048; doi:10.3389/fmicb.2018.00681)
Supplement: Supplementary file 6 [file DataSheet1.PDF]

Supplementary Movie Captions

Movie S1 FtsZ-GFP localization in wildtype cells. Images were acquired every 5 minutes for 126 frames and movie is played at 10 frames per second.

Movie S2 FtsZ-GFP localization in Δ*minE* cells showing division near the growing pole. Images were acquired every 10 minutes for 30 frames and movie is played at 10 frames per second.

Movie S3 FtsZ-GFP localization in Δ*minE* cells showing division near the old pole. Images were acquired every 10 minutes for 30 frames and movie is played at 10 frames per second.

Movie S4 FtsZ-GFP localization in Δ*minE* cells showing multiple ring formation resulting in two successful divisions. Images were acquired every 10 minutes for 30 frames and movie is played at 10 frames per second.

Movie S5 FtsZ-GFP localization in Δ*minE* cells showing multiple ring formation resulting in one failed division and one successful division. Images were acquired every 10 minutes for 30 frames and movie is played at 10 frames per second.
